# Flecainide-Induced Brugada Syndrome in a Patient With Skeletal Muscle Sodium Channelopathy: A Case Report With Critical Therapeutical Implications and Review of the Literature

**DOI:** 10.3389/fneur.2018.00385

**Published:** 2018-05-30

**Authors:** Michele Cavalli, Barbara Fossati, Raffaele Vitale, Elisa Brigonzi, Vito A. G. Ricigliano, Lorenzo Saraceno, Rosanna Cardani, Carlo Pappone, Giovanni Meola

**Affiliations:** ^1^Department of Biomedical Sciences for Health, University of Milan, Milan, Italy; ^2^Department of Neurology, IRCCS Policlinico San Donato, Milan, Italy; ^3^Clinical Arrhythmology and Electrophysiology Department, IRCCS Policlinico San Donato, Milan, Italy; ^4^Laboratory of Muscle Histopathology and Molecular Biology, IRCCS Policlinico San Donato, Milan, Italy

**Keywords:** sodium skeletal muscle channelopathy, *SCN4A*, Brugada syndrome, flecainide, mexiletine

## Abstract

Skeletal muscle sodium channelopathies are a group of neuromuscular disorders associated with mutations in the *SCN4A* gene. Because principal sodium channel isoforms expressed in the skeletal muscles and the heart are distinct one from the other, this condition usually spares cardiac functioning. Nonetheless, evidence on a possible link between skeletal muscle and cardiac sodium channelopathies has emerged in recent years. To date, eight patients bearing pathogenetic mutations in the *SCN4A* gene and manifesting cardiac electrophysiological alterations have been reported in literature. Among these patients, three presented a phenotype compatible with Brugada syndrome. We report the case of a 29-year-old patient affected by non-dystrophic myotonia associated with a p.G1306E mutation in the *SCN4A* gene, who presented symptoms of syncope and palpitation after the introduction of flecainide as an anti-myotonic agent. ECG and ajmaline challenge were consistent with the diagnosis of Brugada syndrome, leading to the implantation of a cardioverter defibrillator. No mutation in causative genes for Brugada syndrome was detected. Mexiletine treatment reduced myotonia without any cardiac adverse events. This case report highlights the clinical relevance of the recognition of cardiac electrophysiological alterations in skeletal muscle sodium channelopathies. The discovery of a possible pathogenetic linkage between skeletal muscle and cardiac sodium channelopathies may have significant implications in patients' management, also in light of the fact that class 1C anti-arrhythmics are potential triggers for life-threatening arrhythmias in patients with Brugada syndrome.

## Background

Brugada syndrome is a dominantly inherited cardiac channelopathy, associated with symptoms of syncope, ventricular arrhythmia and sudden cardiac death (SCD). These symptoms are triggered in time by specific agents, such as fever or the use of sodium channel-blocking drugs. The prevalence is estimated to be worldwide, equal to 1–4 out of 2,000 people. Frequently, patients are initially asymptomatic showing a normal basal ECG, but subsequently they experience aborted SCD or they are diagnosed during a familial screening. A pathogenetic mutation in *SCN5A* gene, coding for cardiac voltage-gated sodium channel (Na_v_1.5), is recognized in 20-25% of cases (1).

Mutations in *SCN4A* gene, encoding for skeletal muscle voltage-gated sodium channel (Na_v_1.4), are known to produce non-dystrophic myotonia, hyper- or hypokalemic periodic paralysis, or congenital myasthenic syndrome (2). Such mutations are usually thought to spare myocardial functioning. This postulate has been questioned by four case reports of patients with T wave alterations and/or QT prolongation in a family with all members affected by paramyotonia congenita (3). Sinus bradycardia with right bundle branch block and QT prolongation were found in a patient affected by hypokalemic periodic paralysis associated with a *SCN4A* mutation (4). More recently, a work by Bissay and colleagues reported the occurrence of Brugada phenocopy in three patients with *SCN4A* gene mutations and in two patients with a mutation variant of uncertain significance (VUS) in the same gene. Notably, none of them showed pathogenetic mutations in *SCN5A* gene (5) (Table 1).

**Table 1:** Reported arrhythmic complications with *SCN4A* mutations.

	**NM diagnosis**	***SCN4A* mutation**	**Symptoms**	**ECG**	**Ajmaline challenge**	**EPS**	***SCN5A* mutation**	**Devices**
Péréon Y et al. (3)	PMC	R1448C	Myotonia, episodic weakness	T wave alteration	n.a.	n.a.	n.a.	None
Péréon Y et al. (3)	PMC	R1448C	Myotonia, muscular hypertrophy	T wave alteration	n.a.	n.a.	n.a.	None
Péréon Y et al. (3)	PMC	R1448C	Myotonia	T wave alteration, long QT	n.a.	n.a.	n.a.	None
Péréon Y et al. (3)	PMC	R1448C	Myotonia	T wave alteration, long QT	n.a.	n.a.	n.a.	None
Maffè et al. (4)	Hypo-PP	R669H	Episodic weakness, syncope	Sinus bradycardia, RBBB, long QT	n.a.	n.a.	n.a.	Temporary PM
Bissay et al. (5)	SCM	L1436P	Myotonia, syncope	Normal	Positive	No inducibility	H558R 1141–3 C>A	ICD
Bissay et al. (5)	SCM	L1436P	Myotonia	Normal	Positive	No inducibility	H558R 1141–3 C>A	None
Bissay et al. (5)	SCM	L1436P	Myotonia	Normal	Positive	No inducibility	–	None
Bissay et al. (5)	–	Q1301del (VUS)	Aborted SCD, myotonia	Brugada pattern I	n.a.	n.a.	–	ICD
Bissay et al. (5)	–	Q1301del (VUS)	Myotonia	Normal	Positive	n.a.	n.a.	None
Present case	SCM (SNEL phenotype)	G1306E	Myotonia, muscular hypertrophy, syncope and palpitation under flecainde	Brugada pattern I under flecainde	Positive	No inducibility	–	ICD

*NM, neuromuscular; EPS, electrophysiological study; PMC, paramyotonia congenita; PM, pacemaker; RBBB, right bundle branch block; Hypo-PP, hypokalemic periodic paralysis; SCM, sodium channel myotonia; SCD, sudden cardiac death; ICD, implantable cardioverter; VUS, variant of uncertain significance; SNEL, severe neonatal episodic laryngospasm*.

## Case presentation

**Figure 1:**
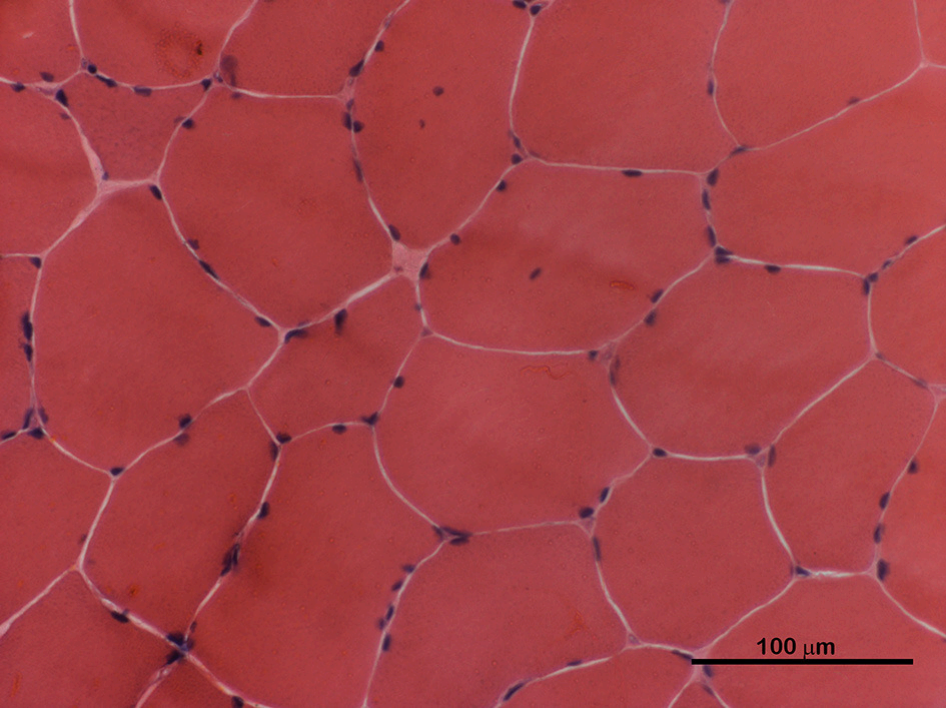
H&E staining of skeletal muscle transverse section performed at the age of 14. Rare atrophic fibers and few centralized nuclei were present.

We report the case of a 29-year-old male affected by non-dystrophic myotonia due to a sporadic p.G1306E mutation in the *SCN4A* gene (6). During neonatal period he presented paroxysms of laryngospasm and respiratory distress, depicting the peculiar features of severe neonatal episodic laryngospasm (SNEL) phenotype (7). These episodes ceased several weeks after birth, consequently to the introduction of an anti-myotonic treatment with mexiletine, 30 mg *quater in die* (qid) per os. Since that intervention, the patients progressively developed diffuse muscular hypertrophy and a myotonia permanens phenotype. Only a few transient adynamic episodes occurred during adolescence. An adjustment of mexiletine dosage up to 400 mg *ter in die* (tid) led to a satisfactory control of the myotonic phenomenon, leaving just a minimal disability. Skeletal muscle biopsy, performed at the age of 14, revealed only moderate fiber size variability, with mild atrophy of type 1 fibers and rare centralized nuclei (Figure 1). A periodical annual cardiological assessment performed with echocardiogram and a 24-h ambulatory ECG showed to be normal.

A therapeutical shift from mexiletine to flecainide was conducted to improve the patient's compliance by reducing his multiple daily administrations. This choice was in agreement with a mutation-targeted approach proposed by Desaphy et al. (8, 9) and clinically reproduced by Portaro et al. (10). Flecainide treatment was started in an outpatient clinical setting and the daily dosage was gradually increased. After reaching a 100 mg *bis in die* (bid) dosage amount, the patient came to our Emergency Department showing symptoms of syncope and palpitation. At the admission the neurological evaluation was normal except for a diffuse muscular hypertrophy, while clinical myotonia was undetectable. A standard 12 lead ECG revealed a prominent coved-type ST elevation with a T wave inversion in the first precordial leads consistent with Brugada pattern type I. Serum electrolytes and other routine blood analyses were normal, except for an awaited hyperCKemia (579 U/l). The flecainide therapy was immediately interrupted and, as a consequence, the coved-type ST elevation noted on the right precordial leads disappeared during the following hours. In parallel with the ECG normalization, myotonia symptoms showed, becoming more and more invalidating as the days passed.

**Figure 2:**
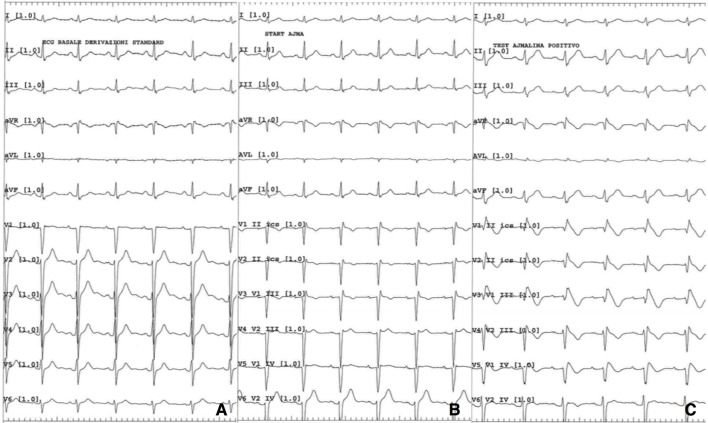
**(A)** Baseline 12 lead ECG; **(B)** ECG while starting ajmaline infusion; **(C)** ECG 5 min after ajmaline infusion starting.

After an appropriate pharmacological wash-out, ajmaline challenge was performed and resulted positive for the appearance of Brugada pattern type I (Figure 2). Programmed ventricular stimulation during electrophysiological study (EPS) was negative for inducible sustained ventricular arrhythmias. Diagnosis of Brugada syndrome was formulated and, given the history of syncope, an implantable cardioverter defibrillator (ICD) was implanted for primary prevention. Next generation sequencing (NGS) genetic analysis with TruSight Cardio Sequencing kit Illumina excluded the presence of sequence variants in *SCN5A* gene and in other 15 genes associated to Brugada syndrome (*ABCC9, CACNA1C, CACNA2D1, CACNB2, GPD1L, HCN4, KCND3, KCNE3, KCNH2, KCNJ8, PKP2, RANGRF/MOG1, SCN1B, SCN3B, TRAPM4*) (11). The analysis revealed two variants of uncertain significance (VUS) in two other genes, namely a p.G678A substitution in *DSG2* and a p.R1013W substitution in *RYR2. DSG2* encodes for desmoglein-2, a cadherin family member, and is related to arrhythmogenic right ventricular dysplasia (ARVD) (12). *RYR2* encodes for ryanodine receptor 2, found in myocardial sarcoplasmatic reticulum, and mutations in its sequence are linked to catecholaminergic polymorphic ventricular tachycardia (CPVT) (13). The same variant in *DSG2* was found in patient's mother, while the one in *RYR2* was present in the father. Both parents were asymptomatic for syncope or palpitation, and their ECGs were normal.

In consideration of the favorable pharmacodynamic profile in Brugada syndrome (14), an oral regimen of hydroquinidine, that is a class 1A anti-arrhythmic drug, was introduced as a potential anti-myotonic agent and was titrated up to 150 mg bid in a few days. This regimen appreciably reduced myotonia, but had to be interrupted due to the appearance of diarrhea. Consequently, the previous mexiletine regimen was restarted. Since then both clinical myotonia and cardiological symptoms disappeared, the ECG remained unaltered and no shock has been delivered by ICD.

## Discussion

Our case suggests that mutations in *SCN4A* may produce *per se* a Brugada phenotype. In the four confirmed cases two different mutations were implicated, therefore currently no genotype-phenotype correlation can be confirmed. The p.G1306E mutation, present in our patient, is located in the sequence coding for the sodium channel inactivation gate and it profoundly alters the channel's electrophysiology (6, 15). Consistently, p.G1306E mutation is associated with severe myotonic phenotypes, such as SNEL (10). We speculate that a severe affection of the Na_v_1.4 physiology could also impair cardiac ion currents and, consequently, have a role in Brugada phenotype expression.

Since a pathogenetic mutation is found only in 20-30% of patients affected by Brugada syndrome, we could not exclude the coexistence of different mutations in the same patient, affecting independently both ion currents in skeletal muscle and in cardiac cells. However, to the best of our knowledge, this is the first report of a potentially arrhythmic condition linked to p.G1306E Na_v_1.4 mutation.

In current literature only two cases of patients with a pathogenetic mutations in *SCN4A* have been reported to manifest syncope, while several subjects presenting positive challenge with class 1C anti-arrhythmics were asymptomatic in the Bissay and colleagues' series. To note, Bissay and colleagues have reported two myotonic patients bearing a VUS in *SCN4A* and presenting Brugada pattern I on baseline ECG; one of the two patients was rescued from a cardiac arrest (5).

In our case, Brugada-associated symptoms showed after the introduction of a twice a day flecainide regimen, initiated in order to mitigate a massive myotonic phenomenon.

While in previous cases only the *SCN5A* gene had been screened, our patient underwent extensive genetic analysis with the NGS technique. This approach revealed the presence of two VUS in two different genes linked to arrhythmic diseases other than Brugada syndrome.

Moreover, two patients who had been reported by Bissay et al. (5) had two polymorphisms in the *SCN5A* gene, which were characterized as possible disease modifying variants (16, 17). If considered altogether, these results suggest a potential role of modifier genes in the determination of Brugada phenotype linked to *SCN4A* mutations.

The putative pro-arrhythmic effect of *SCN4A* mutations is corroborated by the demonstration of Na_v_1.4 expression in human cardiomyocytes (18, 19). However, its real electrophysiological relevance remains to be defined. Notably, reported arrhythmic complications in skeletal muscle sodium channelopathies seem to reproduce the spectrum of electrophysiological alterations associated to *SCN5A* mutations, such as Brugada syndrome and long QT syndrome (20).

It should be remarked that in *SCN4A* gene mutations myotonia is due to a gain-of-function effect in the voltage-gated sodium channel, while in Brugada syndrome there is a loss of function of Na_v_1.5. Moreover, Na_v_1.4 expression in human myocardium appears to be quantitatively marginal, accounting for 1.1–2.6% of all sodium channels (18, 19). Latter assumptions stand against a single-agent effect of Na_v_1.4 in cardiac electrophysiology alterations. Possibly, the Na_v_1.4 contribution to transmembrane ion fluxes in myocardiocytes may become functionally relevant in presence of sodium-channel blocking agents.

Altogether the considerations expressed above suggest an interactive pathogenetic mechanism rather than a direct causative role of Na_v_1.4 dysfunction in Brugada syndrome; in this view, modifier genes may be crucial. We cannot definitively establish if Brugada syndrome and myotonia (or periodic paralysis) could be organ-specific manifestations of the same process.

In a recent study Mannikko et al. found *SCN4A* pathogenetic mutations in four infants out of a cohort of 278 cases of sudden infant death syndrome (SIDS) (21). The authors suggest that acute respiratory dysfunction, which occurs in cases of SNEL, may be the cause of those premature deaths. Given the considerations expressed above, cardiac arrhythmias could be implicated as well, and this hypothesis should be considered for further investigations.

Current data do not seem sufficient to support a systematic screening for Brugada syndrome in all patients with skeletal muscle sodium channelopathy. However, since the symptomatic treatment for myotonia involves the use of sodium channel-blocking agents (22), we suggest the initiation of a class 1C anti-arrhythmic therapy in controlled clinical settings. In this view, we advise the systematic recording of ECG with modified precordial leads to enhance the detection of ST-T changes consistent with Brugada syndrome.

This is the first report of warning symptoms of a potentially life-threatening cardiac condition appearing after the introduction of class 1C anti-arrhythmic therapy in a patient with non-dystrophic myotonia. We do not discourage a flecainide prescription as an anti-myotonic agent in patients with *SCN4A* mutations, given it is effective: however, we advise a cautious approach to this type of treatment. In such cases, flecainide therapy should possibly be introduced also in outpatient clinical settings, even though ECG should be performed before and 3 hours after the first drug administration. Since the steady state with oral flecainide is reached after several days (23), a further ECG should be performed after 1 week of a full dosage therapy.

According to our experience, mexiletine, a class 1B anti-arrhythmic drug, remains a feasible anti-myotonic therapy, in presence of Brugada syndrome. Nonetheless, it should be given under strict cardiological monitoring. In our patient, hydroquinidine was interrupted before reaching full dosage due to gastrointestinal intolerance but showed a clear anti-myotonic effect. Since its pharmacodynamic features, especially the inhibition of K_ito_ current, are favorable in Brugada syndrome, its efficacy as an anti-myotonic agent should be tested in a controlled and larger setting.

In conclusion, emerging data seem to outline an overlapping spectrum between skeletal muscle and cardiac sodium channelopathies. This could include non-dystrophic myotonias, periodic paralysis, long QT and Brugada syndromes. As demonstrated by our case, this has strong implications in the therapeutical management, however confirmatory studies are still needed.

## Ethics statement

The study protocol was conducted according to the principles expressed in the Declaration of Helsinki, the institutional regulation and Italian laws and guidelines. Written informed consents were obtained from the patients for all blood samples and muscle biopsies used in this study.

## Author contributions

MC wrote the manuscript. BF, MC, EB, and RV conducted clinical work-up. RC processed muscle biopsy. MC, EB, and RC made table and figures. MC, BF, EB, LS, VR, and GM reviewed the literature. MC, BF, RV, EB, VR, LS, RC, CP, and GM performed final manuscript review and editing.

### Conflict of interest statement

The authors declare that the research was conducted in the absence of any commercial or financial relationships that could be construed as a potential conflict of interest.
